# Design of Heat-Conductive hBN–PMMA Composites by Electrostatic Nano-Assembly

**DOI:** 10.3390/nano10010134

**Published:** 2020-01-12

**Authors:** Atsushi Yokoi, Wai Kian Tan, Taichi Kuroda, Go Kawamura, Atsunori Matsuda, Hiroyuki Muto

**Affiliations:** 1 Institute of Liberal Arts and Sciences, Toyohashi University of Technology, Toyohashi 441-8580, Japan; yokoi@ion.ee.tut.ac.jp; 2 Department of Electrical & Electronics Information Engineering, Toyohashi University of Technology, Toyohashi 441-8580, Japan; t123231@edu.tut.ac.jp (T.K.); kawamura.go.km@tut.jp (G.K.); matsuda@ee.tut.ac.jp (A.M.)

**Keywords:** electrostatic adsorption, electrostatic assembly, composite, heat-conductive, hBN, PMMA

## Abstract

Micro/nanoscale design of composite materials enables alteration of their properties for advanced functional materials. One of the biggest challenges in material design is the controlled decoration of composite materials with the desired functional additives. This study reports on and demonstrates the homogeneous decoration of hexagonal boron nitride (hBN) on poly(methylmethacrylate) (PMMA) and vice versa. The formation of the composite materials was conducted via a low environmental load and a low-energy-consuming, electrostatic nano-assembly method which also enabled the efficient usage of nano-sized additives. The hBN/PMMA and PMMA/hBN composites were fabricated in various size combinations that exhibited percolated and layer-oriented structures, respectively. The thermal conductivity behaviors of hBN/PMMA and PMMA/hBN composites that exhibited good microstructure were compared. The results showed that microstructural design of the composites enabled the modification of their heat-conducting property. This novel work demonstrated the feasibility of fabricating heat-conductive PMMA matrix composites with controlled decoration of hBN sheets, which may provide a platform for further development of heat-conductive polymeric materials.

## 1. Introduction

Polymer matrix composites with the desired properties attracted the interest of many researchers due to the significant potential for low-cost fabrication of high-performance functional polymeric materials. The composite’s properties can be changed by altering the composition of its organic or inorganic materials. Poly(methylmethacrylate) (PMMA) is a widely used polymeric material due to its unique properties such as good optical clarity, high mechanical strength, and good thermal stability. PMMA also exhibits an amorphous polymer processing dimensional stability that allows it to be used as a host in composite material fabrication [1]. Various PMMA composite materials with desired properties were reported such as functional transparent composites [2,3,4] and infrared (IR) shielding composites [5].

In the rapid development of wearable and high-power electronic devices, the requirement of effective heat-dissipating composite materials is more important than ever [6,7,8]. High-performance electronic devices tend to generate heat rapidly, making heat dissipation a key factor in a device’s design, as heat entrapment will not only affect the performance of the device but also reduce the device’s lifetime. In the electronics industry, synthetic polymers are commonly used in encapsulation, as well as for packaging materials. However, the synthetic polymeric materials used possess low thermal conductivities of 0.1–0.5 W·m^−1^·K^−1^ [9]. Therefore, the development and improvement of heat-conductive polymeric composite materials are crucial to improve the heat-dissipation of electronic devices. Recently, two-dimensional (2D) boron nitride (BN) sheets which possess good thermal conductivity, oxidation resistance, high elastic modulus, and electrical insulation were widely used as 2D micro/nano thermal conductive fillers [9,10,11]. The unique honeycomb-configured *sp*^2^-bonded boron and nitrogen promotes anisotropic thermal conductivity with in-plane and out-of-plane thermal conductivity of 600 and 30 W·m^−1^·K^−1^, respectively [12]. Due to the strong structural correlation of 2D hexagonal boron nitride (hBN) micro and nanosheets with their in-plane thermal conduction, it is imperative to maintain its structure for effective heat conduction. This makes conventional mechanical mixing methods such as mechanical mixing inappropriate as they would destroy the sheet-like structure and hamper the thermal conductivity of the composite, in addition to there being issues when mixing them [1,13]. Furthermore, a high loading amount of more than 50 vol.% is required to generate good thermal conductivity, but this compromises the composite’s mechanical property [13]. In order to preserve the sheet-like structure of hBN sheets, electrostatic nano-assembly (EA) was used in this study. Previously, our group demonstrated the feasibility of an EA method for fabricating various composite materials [14]. The composites were used for applications such as IR shielding [5], optical property-controlled ceramic composite films [15], selective laser sintering [16], mechanical property control of carbon-based alumina composite [17], and fabrication of the negative electrode for rechargeable Fe–air batteries [18]. The EA method not only allows for the homogeneous decoration of additives onto desired primary particles, but also the preservation of the shape and size of the starting materials. Moreover, compared to most reported methods for fabrication of hBN polymeric composite materials such as the chemical vapor deposition method [10], which involve high cost and energy consumption, the EA method is a facile and smart material processing method that involves low environmental load, as well as low energy consumption, which aligns with recently established sustainable development goals. As the fabrication process is carried out in an aqueous medium that is environmentally friendly, chemical contamination could be avoided. Moreover, the mixing is conducted at room temperature with minimal energy consumption compared to high-energy-consuming equipment such as mechanical ball-milling, heated roller mixing [6], and chemical vapor deposition [10]. The EA method also allows the efficient usage of the raw materials, which helps to reduce wastage due to the good recovery rate of the composites while maintaining the shape and structure of the raw materials [14].

In previous reported studies, the effects of the hBN sheet orientation, applied voltage waveform, and composite molding temperature on the electrical breakdown strength and thermal conductivities of only layer-oriented PMMA/hBN composites using hBN with large diameters of 10 and 45 µm as the primary (core) particles were investigated [19,20]. In this study, the feasibility of controlling the microstructure of the pellets using a wider size range combination of PMMA particles and hBN sheets was further demonstrated. A systematic investigation was carried out by adjusting the size of the starting materials (hBN and PMMA), and the microstructural morphologies obtained by decoration of hBN sheets on PMMA particles, and vice versa, were also tabulated. Decoration of hBN sheets onto PMMA particles led to the formation of a percolate-structured microstructure, while decoration of PMMA onto hBN sheets resulted in the formation of a layer-oriented microstructure. The correlation of composite particle assembly, microstructural formation, and heat conductivity of the aforementioned composites was evaluated using IR thermography. The hBN/PMMA composite materials possessed good potential for application as heat-dissipation materials for electronic industries. The results obtained in this work on the controlled design of hBN/PMMA composite materials could be beneficial for further development of heat-conductive polymeric materials.

## 2. Materials and Methods

The experiments were carried out using commercially available poly(methylmethacrylate) (PMMA) particles (average particle diameter of 0.3, 5, and 12 μm, Sekisui Chemical, Tokyo, Japan) and hexagonal boron nitride sheets (average particle size of 0.5 μm, Showa Denko 5, 18 μm Tokyo, Japan, Denki Kagaku, Tokyo, Japan). The polycation and polyanion used were polydiallyldimethyl ammoniumchloride (PDDA) (average molecular weight 100,000 to 200,000, Sigma-Aldrich, Missouri, United States) and polysodium styrenesulfonate (PSS) (average molecular weight 70,000, Sigma-Aldrich, Missouri, MO, United States), respectively. The surfactant used for the initial coating onto PMMA was sodium deoxycholate (SDC). After that, an alternative layer of PDDA/PSS/PDDA was coated onto PMMA to induce positive zeta-potential-modified PMMA primary particles. Similarly, for the hBN sheets, the surface charge was firstly modified using SDC, followed by PDDA and PSS, to obtain a negative zeta-potential surface. Then, the oppositely charged PMMA particles (positive) and hBN nanosheets (negative) in aqueous solution were mixed and stirred to obtain the electrostatically assembled hBN/PMMA composite particles. The coverage amount of additive particles (hBN or PMMA) on intended primary particles (PMMA or hBN) was adjusted in vol.% according to previous reported work [14].The suspension was then dried to obtain the composite powder. For the heat distribution evaluation of the hBN/PMMA composite obtained, the composite powders were pressed into pellets using a hot press with a pressure of 100 MPa at 200 °C for 15 min. Prior to pressing, 50 μL of methyl methacrylate (MMA) was added to promote the bonding of the main chain and to improve the mold-ability. The pressed pellets were then annealed at 80 °C for 15 min. Six different combinations of hBN with PMMA consisting of different sizes, as mentioned above, were fabricated for comparison. The morphological structures of the composites obtained were observed using an S-4800 field-emission scanning electron microscope (FE-SEM, Hitachi S-4800, Tokyo, Japan). For the cross-sectional observation, the composite pellets were cut into half and polished using a surface grinder prior to SEM observation. The heat distribution properties of the nanocomposite pellets were evaluated using infrared thermography (Testo corporation, Testo 881-1, Lenzkirch, Germany).

## 3. Results and Discussion

**Figure 1:**
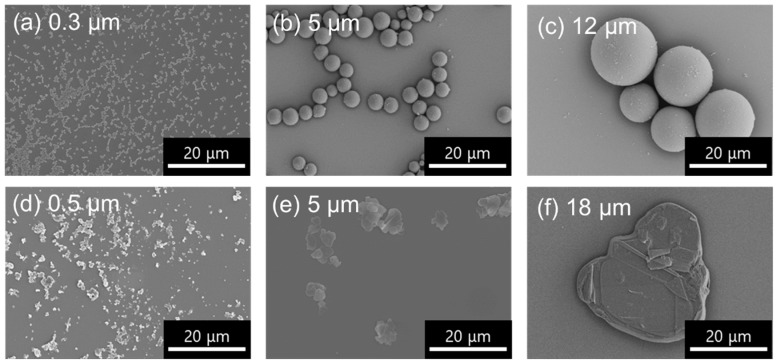
SEM images of the starting materials used for hexagonal boron nitride (hBN)/poly(methylmethacrylate) (PMMA) composite formation. PMMA particles with different sizes of (**a**) 0.3, (**b**) 5, and (**c**) 12 µm, and hBN sheets with different sizes of (**d**) 0.5, (**e**) 5, and (**f**) 18 µm were used.

The SEM images of the starting materials used for this study are shown in Figure 1. The spherical PMMA particles, with average sizes of 0.3, 5, and 12 µm, are shown in Figure 1a–c, respectively. On the other hand, the SEM images of the sheet-like hBN, with average sizes of 0.5, 5, and 18 µm, are shown in Figure 1d–f respectively. Depending on the size of the primary particle (either PMMA or hBN), smaller-sized secondary/additive particles (hBN or PMMA) were used for electrostatic decoration on the surface of the primary particle. For example, smaller-sized hBN sheets (0.5 µm) were used for electrostatic assembly onto the surface of larger spherical PMMA particles (12 µm).

**Figure 2:**
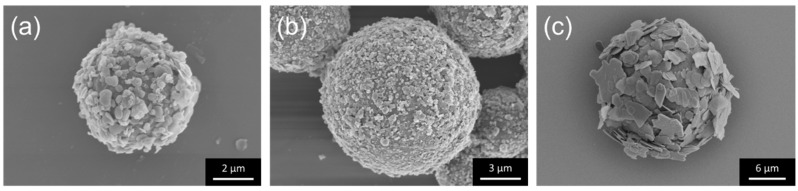
SEM images of the hBN/PMMA (PMMA core) composite particles obtained after electrostatic nano-assembly method using various sizes combination of (**a**) 0.5-µm hBN on 5-µm PMMA, (**b**) 0.5-µm hBN on 12-µm PMMA, and (**c**) 5-µm hBN on 12-µm PMMA.

In this study, two different composites with either spherical PMMA particles or two-dimensional hBN sheets were used as the primary particle (core). Different size combinations of PMMA and hBN were investigated systematically. Firstly, decoration of sheet-like hBN with an average diameter of 0.5 or 5 µm was carried out on PMMA with an average diameter of 5 or 12 µm. The hBN/PMMA composite particles obtained, with the combinations of (0.5 µm) hBN/PMMA (5 µm), (0.5 µm) hBN/PMMA (12 µm), and (5 µm) hBN/PMMA (12 µm) are shown in Figure 2. Regardless of the hBN sheet size, homogeneous decoration of hBN sheets on PMMA particles was obtained. A good surface coverage of 0.5-µm hBN on both 5- and 12-µm PMMA was observed, as shown in Figure 2a,b, respectively. Figure 2c further demonstrates the feasibility of decorating 12-µm PMMA particle with larger hBN having an average diameter of 5 µm.

**Figure 3:**
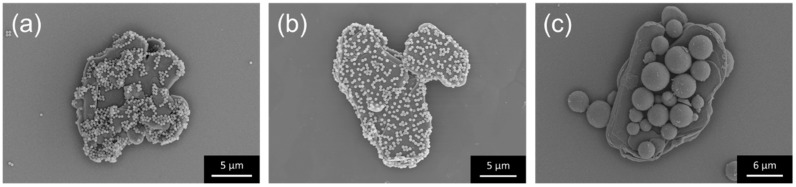
SEM images of the PMMA/hBN (hBN core) composite particles obtained after electrostatic assembly, using various combinations: (**a**) 0.3-µm PMMA on 5-µm hBN sheets, (**b**) 0.3-µm PMMA on 18-µm hBN sheets, and (**c**) 5-µm PMMA on 18-µm hBN sheets.

Subsequently, hBN sheets with different sizes, 5 and 18 µm, were used as the primary matrix (core) while PMMA with different average particle sizes of 0.3 and 5 µm was used as the additives in the electrostatic nano-assembly. PMMA/hBN composite particles with the combinations of (0.3 µm) PMMA/hBN (5 µm), (0.3 µm) PMMA/hBN (18 µm), and (5 µm) PMMA/hBN (18 µm) are shown in Figure 3. PMMA particles were observed to be homogeneously distributed on the surface of hBN sheets. The morphological observations of the composites obtained in Figure 2 and Figure 3 indicated that an electrostatic nano-assembly method could be used for the homogeneous assembly of hBN sheets on PMMA particles or vice versa.

**Figure 4:**
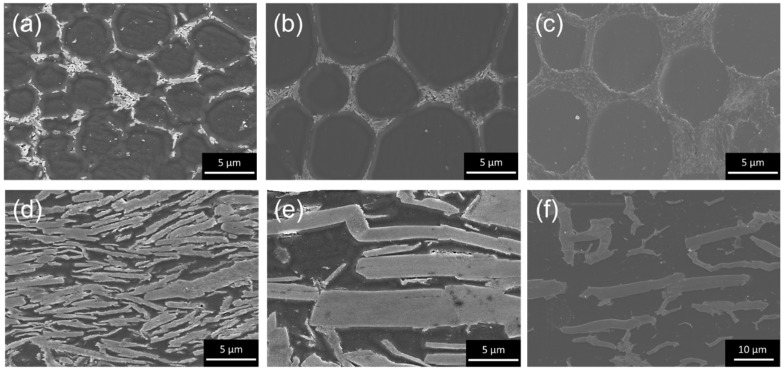
Cross-sectional SEM images of the respective hBN/PMMA composite pellets obtained after hot-pressing, using the following composite powders: (**a**) 0.5-µm hBN on 5-µm PMMA, (**b**) 0.5-µm hBN on 12-µm PMMA, (**c**) 5-µm hBN on 12-µm PMMA, (**d**) 0.3-µm PMMA on 5-µm hBN sheets, (**e**) 0.3-µm PMMA on 18-µm hBN sheets, and (**f**) 5-µm PMMA on 18-µm hBN sheets.

**Figure 5:**
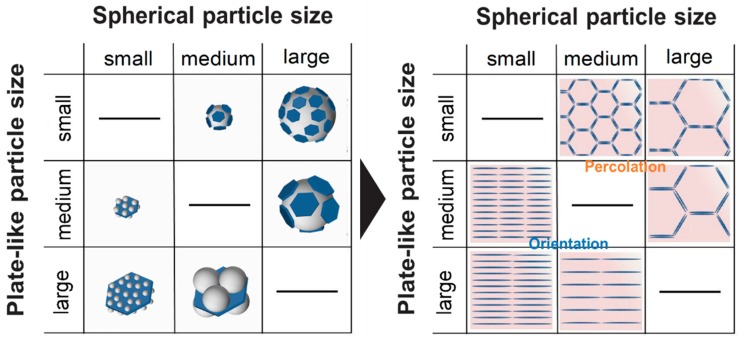
Schematic illustrations showing the morphologies of hBN/PMMA and PMMA/hBN composite particles with the corresponding microstructures obtained.

Subsequently, the hBN/PMMA and PMMA/hBN composite powders shown in Figure 2 and Figure 3 were hot-pressed into pellet and grinded for cross-sectional SEM observation. The cross-sectional SEM images of the composite pellets obtained are shown in Figure 4. Interestingly, hBN/PMMA and PMMA/hBN composite pellets exhibited different microstructural morphologies of percolate structure or layer-oriented structure, respectively. The percolation of hBN on the surface of the PMMA grain boundaries was clearly observed as shown in Figure 4a–c. The degree of percolation of hBN within the PMMA matrix increased with the size of PMMA particles used. This was due to the lower overall surface area available for the electrostatic adsorption of hBN sheets when larger PMMA particles were used. This promoted a denser decoration of hBN on the PMMA surface, generating an interconnected percolation structure. The difference can be observed by comparing Figure 4a,b, where similarly sized 0.5-µm hBN sheets were decorated on PMMA particles with different diameter sizes of 5 and 12 µm, respectively. Similar phenomena were also observed in our previous work involving the decoration of carbon nanoparticles on alumina granules [17]. The highest percolation degree was observed using the composite powders with 5-µm hBN sheets as additives with 12-µm PMMA as the primary particles. On the other hand, disconnected, sheet-like layered microstructures were observed using PMMA-decorated hBN sheets as shown in Figure 4d–f. The interlayer distance between hBN layers was observed to increase when larger PMMA particles were used, as shown in the SEM images of Figure 4e,f, where similar hBN sheets with an average diameter of 18 µm were used. Meanwhile, the hBN sheets were observed to exhibit a more random orientation within the matrix when composites that consisted of smaller PMMA particles decorated on smaller hBN sheets were used compared to those on larger hBN sheets as shown in Figure 4d,e. In previous studies, the orientation probability of layer-oriented PMMA/hBN was altered by changing the size of PMMA particles; decoration of larger PMMA particles on hBN sheets led to irregular hBN sheet direction (larger angle difference) in the composite matrix as compared to when smaller PMMA particles were used [19,20]. Thus, this study shows that the orientation could also be affected by changing the size of the hBN sheets. These results demonstrated that the interlayer distance, as well as the orientation of the layer-structured PMMA/hBN composites, could be controlled by altering the size of PMMA particles decorated on hBN sheets during the assembly step. The schematic illustration in Figure 5 shows the inter-correlation of the hBN/PMMA and PMMA/hBN composite morphologies, with the corresponding microstructures obtained after hot-pressing.

**Figure 6:**
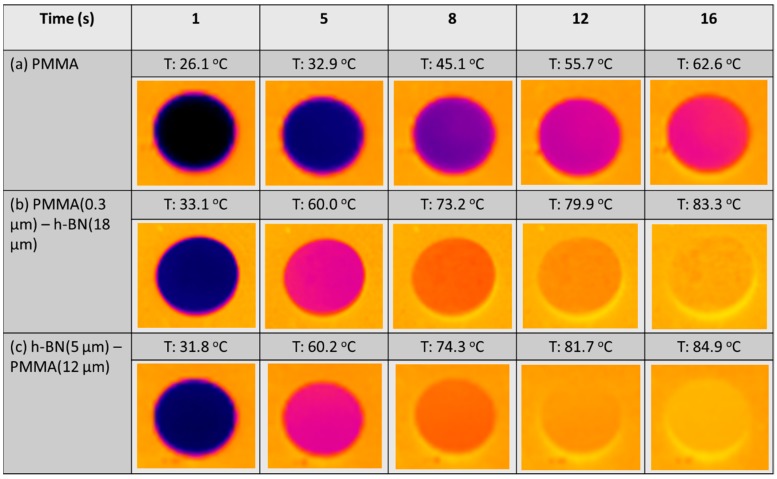
Thermograph images of (**a**) PMMA, (**b**) PMMA (0.3 µm)/hBN (18 µm). and (**c**) hBN (5 µm)/PMMA (18 µm) composite pellets after infrared thermography irradiation.

To gain insight into the thermal conduction properties, infrared thermography comparison was carried out using the pellets fabricated using only PMMA (12 µm), (0.3 µm) PMMA/hBN (18 µm), and (5 µm) hBN/PMMA (12 µm). The composite pellets of (0.3 µm) PMMA/hBN (18 µm) and (5 µm) hBN/PMMA (12 µm) were chosen due to the ordered microstructure obtained compared to others. The thermograph images obtained are shown in Figure 6. From the thermograph image comparison, the heat signatures of the composite pellets, (0.3 µm) PMMA/hBN (18 µm) and (5 µm) hBN/PMMA (18 µm), were observed to be higher than the PMMA pellets. After 16 s, the temperature exhibited by the (0.3 µm) PMMA/hBN (18 µm) and (5 µm) hBN/PMMA (18 µm) pellets was 83.3 and 84.9 °C, respectively, while that of the PMMA pellet was 62.6 °C. These results demonstrated that hBN incorporation into a PMMA matrix enhanced the heat conductance of the composite pellets.

**Figure 7:**
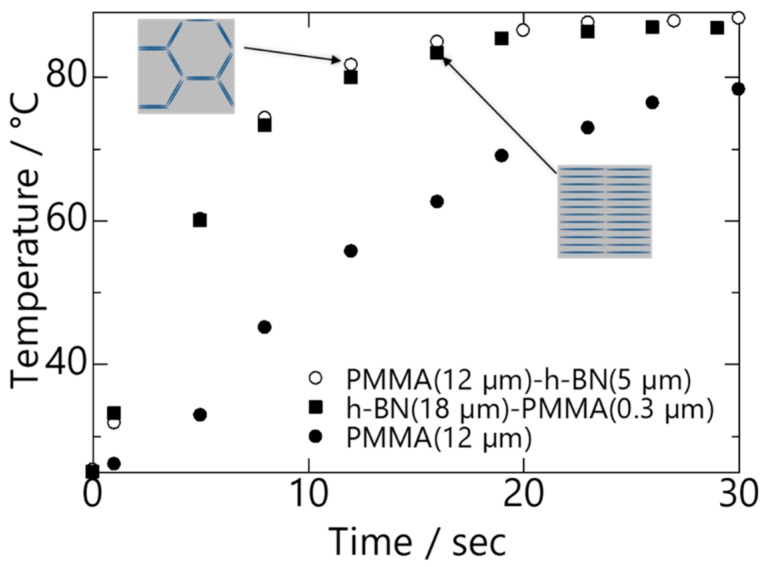
Comparison of temperature change against time for PMMA, layer-oriented PMMA/hBN, and percolation-oriented hBN/PMMA composites.

A graph showing plots of temperature change against time for PMMA (18 µm), (0.3 µm) PMMA/hBN (18 µm). and (5 µm) hBN/PMMA (18 µm) is shown in Figure 7. The results show that heat conduction reached a plateau after 20 s for all the samples, with the composite pellets achieving temperature higher than 80 °C. Although both composite pellets demonstrated almost comparable heat conductivity, it is important to note that the amount of hBN added into the composites differed. The amount of hBN present in (0.3 µm) PMMA/hBN (18 µm) and (5 µm) hBN/PMMA (12 µm) was 57 vol.% and 26 vol.%, respectively. This indicates that, despite having half the volume of hBN incorporated, the (5 µm) hBN/PMMA (18 µm) composite pellet that had a percolated structure exhibited heat-conducting properties comparable to the layer-oriented structure of the (0.3 µm) PMMA/hBN (18 µm) composite pellet. Due to the higher heat conductivity of the hBN sheets in the *a*- and *b*-axis, the percolated microstructure allowed better heat conductivity in the PMMA matrix [21]. The calculated heat conductivity for (0.3 µm) PMMA/hBN (18 µm) and (5 µm) hBN/PMMA (12 µm) was 1.27 and 1.42 W/m∙K, respectively. Zhi et al. reported an approximately three-fold increase in thermal conductivity from 0.17 to 0.50 W/m∙K using a 10 vol.% BN nanotube/PMMA composite film [22]. In comparison with the work reported by Pullanchiyodan et al., using Ag-decorated BN nanosheets in PMMA, they reported a thermal conductivity of 1.48 W/m∙K by incorporating 35 vol.% of this hybrid filler into PMMA matrix [23]. Therefore, the thermal conductivity of 1.42 W/m∙K obtained in this study using percolate-structured hBN/PMMA (26 vol.%) demonstrates promising potential, given the simplicity and good reproducibility of this method. The interconnectivity and interaction of hBN sheets were the determining factors in this heat-conducting property, and the percolated structure of hBN/PMMA composite provided a thermal conductive pathway compared to the layer-structured PMMA/hBN composite. Similar findings were also reported by Mosanenzadeh et al. in their study on the thermal behavior of ordered and random hBN networks using different types of polymers [24]. In addition, the higher content of hBN within the PMMA matrix reduced the mechanical properties drastically, as hBN acted as a defect in the polymer matrix [13]. A better approach would be to precisely design the composite powders to promote a particulate interaction, which not only promotes the efficient use of hBN but also does not compromise its mechanical properties [24].

This study demonstrated that the microstructure of an hBN/PMMA composite is crucial to its property of heat conductivity. Therefore, controlled design of composite formation is crucial using a bottom-up fabrication approach, which is not achievable using the conventional mixing method.

## 4. Conclusions

The controlled microstructure formation of hBN/PMMA and PMMA/hBN composite pellets was systematically investigated using an electrostatic nano-assembly method. The composite pellets of both hBN/PMMA (PMMA core) and PMMA/hBN (hBN core) composites exhibited promising heat-conducting properties. By varying the sizes of the starting materials in the composite powder design, percolate-structured hBN/PMMA and layer-structured PMMA/hBN composite pellets were fabricated from the composite powders obtained. With hBN composition at 26 vol.% for the percolate-structured hBN/PMMA composite compared to 57 vol.% for the layer-structured PMMA/hBN composite, both composites demonstrated almost comparable thermal conductivity behavior during infrared thermography characterization. The thermal conductivity obtained for (0.3 µm) PMMA/hBN (18 µm) and (5 µm) hBN /PMMA (12 µm) was 1.27 and 1.42 W/m∙K, respectively. The results indicated that the percolate-structured hBN/PMMA composite pellet (half the hBN content in comparison to PMMA/hBN composite) exhibited higher thermal conductivity compared to the layer-structured PMMA/hBN composite pellet due to a better heat-conducting route in the percolated structure. This study demonstrated the importance and feasibility of microscale structural design of composite materials in achieving the desired properties such as improved thermal conductivity. The results obtained in this work demonstrate the feasibility of a controlled fabrication of heat-conductive PMMA matrix composites by controlled decoration of hBN sheets and provide a platform for further development of heat-conductive polymeric materials. This electrostatic nano-assembly method not only enables controlled design of the composite particles, which is not achievable using conventional mixing methods, but it also has a low environmental load and low energy consumption. Smart fabrication processes with a low environmental load and low-energy-consumption methods are indispensable as we move toward a sustainable society, and electrostatic nano-assembly is one of the feasible methods in heat-conductive material design.
